# Pregnancy period and early-life risk factors for inflammatory bowel disease: a Northern Finland birth cohort 1966 study

**DOI:** 10.1186/s12889-024-18549-z

**Published:** 2024-04-15

**Authors:** Timo M. Blomster, Olli-Pekka Koivurova, Ritva Koskela, Karl-Heinz Herzig, Nicholas J. Talley, Jukka Ronkainen

**Affiliations:** 1 Department of Internal Medicine, Institute of Clinical Medicine, University of Oulu, Oulu, Finland; 2 Terveystalo Oulu, Medical Center, Oulu, Finland; 3 Research Unit of Biomedicine and Internal Medicine, Biocenter of Oulu, Medical Research Center, University of Oulu and Oulu University Hospital, Oulu, Finland; 4 Department of Gastroenterology and Metabolism, Poznan University of Medical Sciences, Poznan, Poland; 5 School of Medicine and Public Health, Hunter Medical Research Institute, University of Newcastle, NHMRC Centre of Research Excellence in Digestive Health, Newcastle, Australia; 6 Primary Health Care Center, Lapland Welfare District, Tornio, Finland; 7 Research Unit of Population Health, University of Oulu, Faculty of Medicine, FIN-90014 Oulu, P.O. Box 5000, Finland

**Keywords:** Cohort studies, Crohn’s disease, Early life, Epidemiology, Inflammatory bowel diseases, Smoking, Ulcerative colitis

## Abstract

**Background:**

The pathogenesis of inflammatory bowel disease (IBD) has not been fully elucidated. The aim of this study was to analyze the pregnancy period, perinatal period, and infancy period risk factors for IBD in a well-characterized birth cohort from Northern Finland.

**Methods:**

The Northern Finland Birth Cohort 1966 (NFBC1966) population comprises mothers living in the two northernmost provinces of Finland, Oulu, and Lapland, with dates of delivery between Jan 1st and Dec 31st, 1966 (12 055 mothers, 12 058 live-born children, 96.3% of all births during 1966). IBD patients were identified using hospital registries (from 1966 to 2020) and Social Insurance Institution (SII) registry reimbursement data for IBD drugs (from 1978 to 2016). The data were analyzed by Fisher’s exact test and logistic regression.

**Results:**

In total, 6972 individuals provided informed consent for the use of combined SII and hospital registry data. Of those, 154 (2.1%) had IBD (113 [1.6%] had ulcerative colitis (UC), and 41 (0.6%) had Crohn’s disease (CD)). According to multivariate analysis, maternal smoking > 10 cigarettes/day during pregnancy was associated with a nearly 6-fold increased risk of CD in the offspring (OR 5.78, 95% CI 1.70–17.3). Breastfeeding (OR = 0.18, 95% CI 0.08–0.44) and iron supplementation during the first year of life (OR = 0.43, 95% CI 0.21–0.89) were negatively associated with CD.

**Conclusions:**

Smoking during pregnancy was associated with the risk of CD while Breastfeeding and oral iron supplementation at infancy were negatively associated with the risk of CD later in life.

## Introduction

Crohn’s disease (CD) and ulcerative colitis (UC) are chronic inflammatory bowel diseases (IBDs); these conditions have a negative impact on health-related quality of life (HRQoL), are linked to serious comorbidities and complications, including cancer [1], and impose a major economic burden on individuals and society. However, the pathogenesis of IBD is still unclear. It is thought that environmental factors in combination with a genetic predisposition, a dysregulated immune system and an altered gut microbiota are involved in the pathogenesis of IBD [2]. The incidence and prevalence of IBD have been increasing in recent decades worldwide, although the incidence of IBD may now be stabilizing in Western countries [3]. IBD has an exceptionally high prevalence in Scandinavia and North America, and in Europe, the incidence of IBD increased up to 100-fold between 1930 and 2008 [4]. One of the highest incidences rates of IBD in the world has been reported in Finland, especially for ulcerative colitis (UC), estimated to be 34.0 per 100,000 for IBD and 24.8 for UC [5]. The relatively rapid increase in IBD incidence during recent decades suggests that environmental factors play a more important role than genetic factors, although the strongest risk factor for IBD development is a first-degree relative with IBD [6]. This hypothesis is supported by the fact that immigrants and their offspring from countries with a low incidence of IBD have an increased incidence of IBD after moving to a country with a high incidence of IBD [7]. There is intense interest in environmental factors such as diet, which may have an impact on the intestinal bacterial flora [8, 9]. Since the intestinal microbiome starts developing rapidly within the first year of life [10], it is intriguing to speculate that early childhood and perinatal events modify the microbiome and thus may influence future IBD risk.

Several case‒control studies have reported perinatal or early childhood risk factors for IBD, but the results are conflicting [11–15]. Usually, these studies include standard environmental factors such as rural vs. urban living environments, smoking habits of pregnant mothers, mode of delivery, breastfeeding, vaccinations and exposure to antibiotics during pregnancy or early childhood. A recent meta-analysis showed a protective effect of breastfeeding against the development of IBD, with the lowest risk for those who were breastfed for at least 12 months [16]. Antibiotic use in early childhood has been linked to an increased risk of CD but not UC [17]. Living in an urban environment has been associated with an increased risk of IBD [18]. Some studies suggest that polio vaccinations are associated with an increased risk for IBD [12, 19], but a meta-analysis from 2014 concluded that these results should be interpreted with caution because of study heterogeneity [20].

The aim of this study was to analyze the pregnancy period, perinatal period, and infancy period risk factors for IBD in a well-characterized birth cohort from Northern Finland. We hypothesized that the pregnancy period and perinatal circumstances may be important for determining the risk of IBD.

## Methods

### Study population

**Fig. 1:**
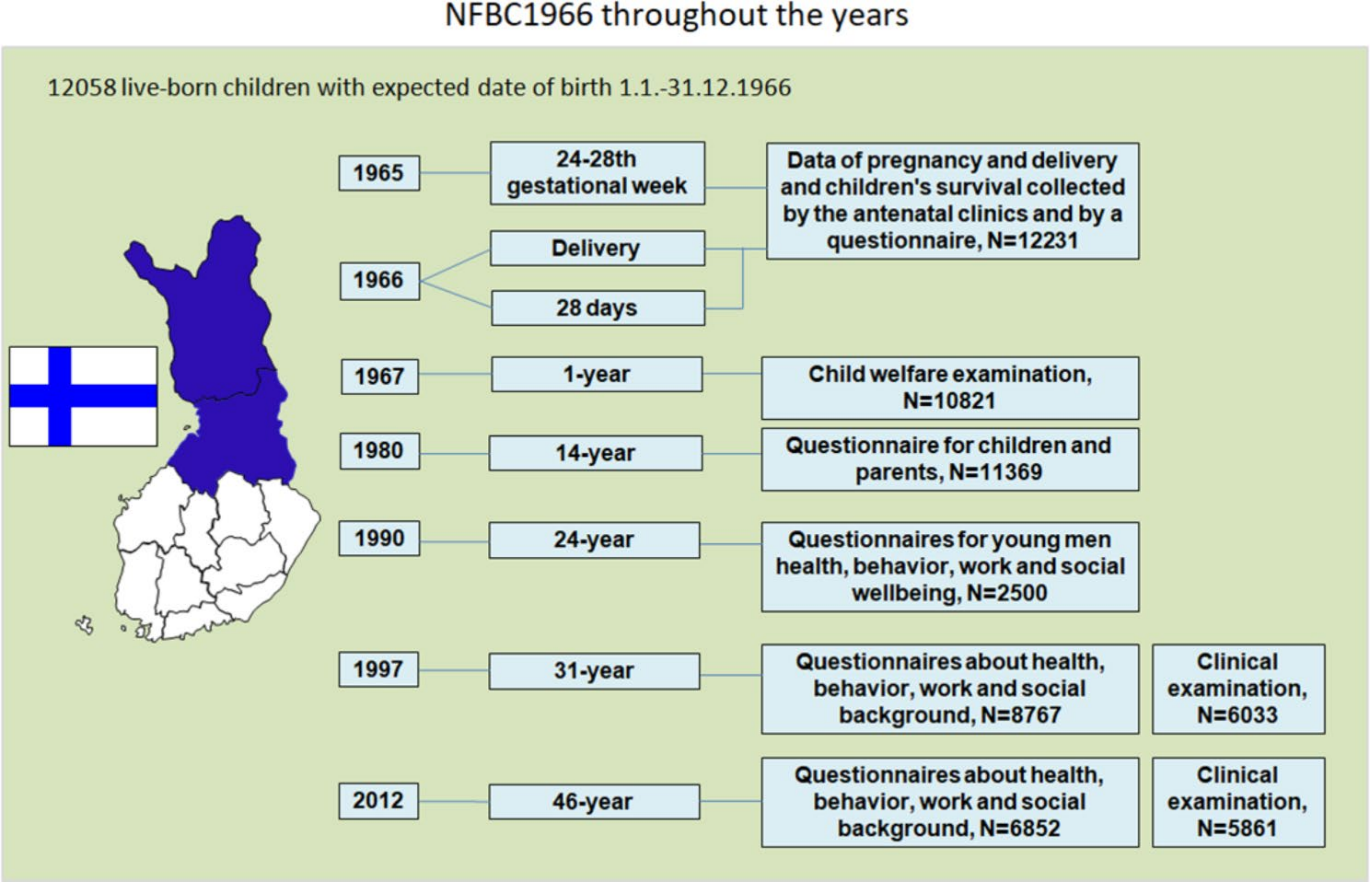
Flow diagram of the study selection process

The prospectively collected Northern Finland Birth Cohort 1966 (NFBC1966 In the list of references: University of Oulu: Northern Finland Birth Cohort 1966. University of Oulu. http://urn.fi/urn:nbn:fi:att:bc1e5408-980e-4a62-b899-43bec3755243) is a longitudinal research program aimed at promoting the health and well-being of the population (http://www.oulu.fi/nfbc/). The population comprises mothers and their offspring living in the two northernmost provinces of Finland, Oulu and Lapland, with expected dates of delivery between Jan 1st and Dec 31st, 1966 (12 055 mothers, 12 231 children, 12 058 live born, 96.3% of all births during 1966 in that area). The history of NFBC1966 is illustrated in Fig. 1.

### Data collection and variables

Information about family history, social relationships, the environment, and parents’ health during pregnancy was collected from antenatal clinics, hospital registers and postal questionnaires. The pregnancy period questionnaire was completed from the 24th to 28th gestational week; if that was not possible, the questionnaire was completed later during the pregnancy or after delivery (10% of mothers). Obstetric data were collected during the perinatal period in delivery hospitals. Information about infant health, breastfeeding status, vaccination status, consumption of antibiotics, and vitamin D and iron supplementation status until the age of one year was collected from child health center visits.

Urban residence included people who lived in towns, and rural residents were people who lived in counties or country communes. The farm animals included cattle, poultry, pigs, lambs, horses, and minks. Individuals with higher social class were defined as parents who had higher administrative or professional occupations [21]. Subjects were divided into those who smoked less than or more than 10 cigarettes/day during pregnancy. Information about vitamin D and iron supplementation, use of antibiotics during pregnancy or the first year of life and vaccination data were collected from the questionnaires described above [21, 22]. Vitamin D and iron supplementation were defined as regular supplementation during the first year of life (response alternatives: not at all, irregularly or regularly according to the 1-year health control questionnaire).

IBD patients were identified by using the International Classification of Diseases (ICD-8, ICD-9, and ICD-10) codes of the Finland hospital outpatient and inpatient registries (from 1966 to 2020). The IBD patients were also identified using reimbursement data from the Social Insurance Institution (SII) of Finland for IBD drugs (from 1978 to 2016). If patients have a private or workplace insurance/subvention, they do not need to apply for SII reimbursement for their medications although they have a valid medication; thus, the hospital registries are the basis of diagnosis in the study. However, subjects with just one inpatient or outpatient visit for an IBD diagnosis were excluded to ensure validity. The SII has records from all individuals who are entitled to reimbursement drugs using treatment for IBD since 1978. Between 1978 and 1993, UC and CD had different reimbursement codes, but since 1994, all IBD patients have been reimbursed under the same code. As each Finnish citizen has their own unique personal social security number, this enabled us to identify persons who had IBD. Hospital registers were also used to separate IBD patients as UC or CD patients by using these ICD codes since this was not possible because of the use of SII reimbursement data after 1994. Individuals who had unclassified or intermediate colitis were included in the UC group.

The study was approved by the Ethics Committee of the Northern Ostrobothnia Hospital District, and combined data were analyzed only from the individuals who provided written informed consent for the use of both the SII registration and hospital registration records.

### Statistical methods

The data were analyzed by Fisher’s exact test and logistic regression. Clinically important and statistically significant variables in univariate analyses were introduced into the regression models, and the goodness of fit of the models was defined. The data were analyzed with the Intercooled STATA11 program [23].

## Results

There were 6972 out of 10 329 individuals available at the time of the 46-year questionnaires who gave informed consent to combine the SII and hospital registry data. Of those 154 (2.2%) had IBD, 113 (1.6%) had UC, and 41 (0.6%) had CD. The prevalence of IBD in all subjects (*n* = 12,058) was 1.5% according to hospital registry data only. Most patients (*n* = 128) were found from SII reimbursement registries. Reimbursement was continued for all but 2 subjects through 2016. An additional 26 patients were found from hospital registries only.

**Fig. 2:**
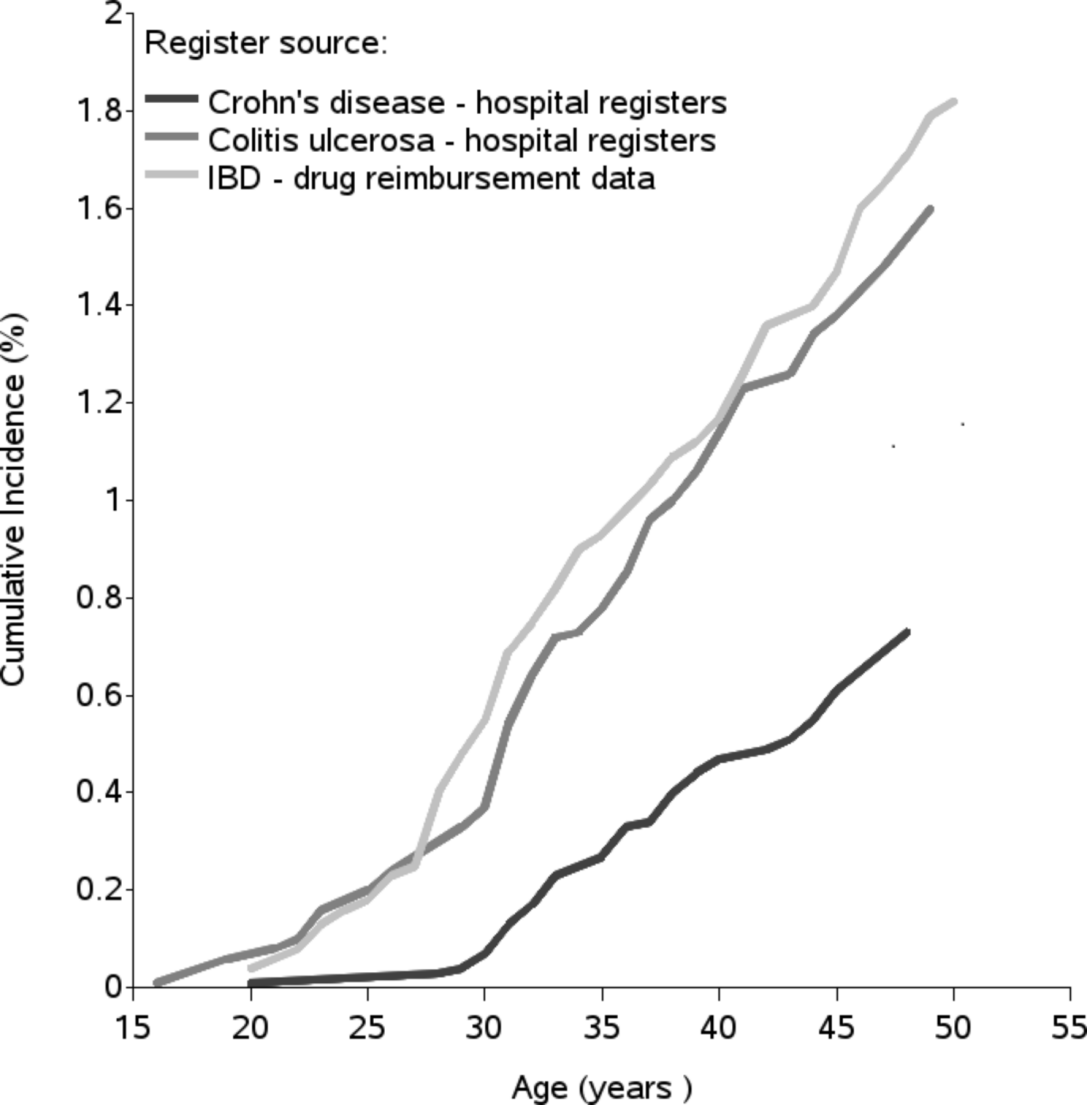
Risk of bias summary for randomized controlled study (*n* = 1)

IBD was diagnosed mainly in adulthood, and the incidence of UC began to increase after 20 years of age, while that of CD began to increase after 30 years of age (Fig. 2).

According to univariate analyses, maternal age, parity, low birth weight, Apgar score, mode of delivery, household farm animals, living in an urban area and social class were not associated with either UC or CD (data not shown). UC in the offspring was associated with UC in the father (5/113 vs. 74/6859, *p* = 0.009), but this was not observed in the CD patients. IBD in mothers was not associated with IBD in the offspring (data not shown).

According to univariate analyses, the consumption of antibiotics during pregnancy was associated with the risk of CD but not UC [UC (8/609 vs. 96/5727) *p* = 0.616; CD 9/609 vs. 28/5727) *p* = 0.007]. According to univariate analyses, regular oral iron supplementation during the first year of life was negatively associated with CD (Table 1). Consumption of antibiotics during the first year of life or regular or irregular use of vitamin D during the first year of life were not associated with UC or CD.

**Table 1:** Infancy period (within 1 year) risk factors for Crohn’s disease (CD) and ulcerative colitis (UC) in crude analysis

	CD		UC	
	*n* = 41, %	OR (95% CI)	*n* = 113, %	OR (95% CI)
Breast feeding at least 1 month*				
Yes, *n* = 2996	11, 0.37	0.18 (0.08–0.45)	54, 1.80	1.18 (0.53–2.61)
No, *n* = 457	9, 1.97		7, 1.53	
Vitamin D supplementation**				
Yes, *n* = 5517	29, 0.53	0.46 (0.21–1.02)	95, 1.72	0.93 (0.52–1.69)
No, *n* = 710	8, 1.13		13, 1.83	
Iron supplementation***				
Yes, *n* = 3603	13, 0.36	0.41 (0.20–0.81)	56, 1.53	0.80 (0.54–1.18)
No, *n* = 2488	22, 0.88		48, 1.93	
**DTP vaccination**				
Yes, *n* = 5647	29, 0.51	0.45 (0.21–1.00)	92, 1.63	0.72 (0.42–1.23)
No, *n* = 712	8, 1.12		16, 2.25	
**Polio vaccination**				
Yes, *n* = 5038	26, 0.52	0.58 (0.29–1.18)	81, 1.61	0.77 (0.49–1.20)
No, *n* = 1247	11, 0.88		26, 2.00	
**Smallpox vaccination**				
Yes, *n* = 962	7, 0.73	1.25 (0.55–2.86)	22, 2.29	1.51 (0.93–2.44)
No, *n* = 4977	29, 0.58		76, 1.53	
**Antibiotics during first year**				
Yes, *n* = 3218	16, 0.50	0.75 (0.38–1.46)	53, 1.65	0.92 (0.63–1.36)
No, *n* = 2862	19, 0.66		51, 1.78	

DTP, diphtheria, tetanus and pertussis vaccination ≥ 3 times

polio vaccination *≥ 2 times*

*Information available, *n* = 3453

** Regular vitamin D supplementation

*** Regular iron supplementation

Diphtheria, tetanus, and pertussis vaccination (DTP) in children vaccinated ≥ 3 times and polio vaccination ≥ 2 times were not associated with the risk for CD or UC (Table 1). Breastfeeding (data available only for 3453 subjects) was negatively associated with CD (Table 1).

According to multivariate logistic regression analysis, maternal smoking of > 10 cigarettes/day during pregnancy was associated with a risk of CD in their offspring later in life (Table 2). Breastfeeding and regular oral iron supplementation during the first year of life were negatively associated with CD (Table 3). According to multivariate logistic regression analysis, neither DTP vaccination nor polio vaccination had a negative influence on IBD risk (Table 3).

**Table 2:** Maternal risk factors during pregnancy for Crohn’s disease (CD) and ulcerative colitis (UC) by logistic regression

	Crude (OR with 95% CI)		Adjusted (OR with 95% CI)	
	CD	UC	CD	UC
	*n* = 41	*n* = 113		
**Smoking**	***n***	***n***		
no (ref) (*n* = 5 967)	34	100		
1–10 cigarettes (*n* = 582)	*4* 1.21 (0.43–3.41)	*11* 1.17 (0.62–2.19)	1.44 (0.50–4.15)	1.20 (0.62–2.33)
> 10 cigarettes (*n* = 103)	*3* 5.23 (1.58–17.3)	*2* 1.20 (0.29–4.93)	5.78 (1.70–19.7)	1.36 (0.33–5.62)
**Antibiotic use**	***n***	***n***		
no (ref) (*n* = 5727)	28	96		
yes (*n*=609)	*9* 3.05 (1.43–6.50)	*8* 0.88 (0.44–1.75)	2.33 (0.95–5.72)	0.91 (0.44–1.89)

Mother’s smoking through pregnancy and use of antibiotics during pregnancy. Adjusting for infant’s iron supplementation and DPT and polio vaccination

**Table 3:** Infancy period (within 1 year) risk factors for Crohn’s disease (CD) and ulcerative colitis (UC) by logistic regression

	CD, *n* = 41, OR (with 95% CI)	UC, *n* = 113, OR (with 95% CI)
Breast feeding* No, (ref) *n* = 448 Yes, *n* = 2985	*n* = 9 *n* = 11, 0.18 (0.08–0.44)	*n* = 7 *n* = 54, 1.05 (0.47–2.32)
Iron supplementation** No, (ref) *n* = 2 488 Yes, *n* = 3 606	*n* = 22 *n* = 13, 0.43 (0.21–0.89)	*n* = 48 *n* = 56, 0.72 (0.48–1.09)
DTP vaccination No, (ref) *n* = 712 Yes, *n* = 5 647	*n* = 8 *n* = 29, 0.55 (0.23–1.34)	*n* = 16 *n* = 92, 0.74 (0.42–1.32)
Polio vaccination No, (ref) *n* = 1 247 Yes, *n* = 5 038	*n* = 11 *n* = 26, 0.66 (0.31–1.44)	*n* = 26 *n* = 86, 0.82 (0.51–1.31)

*Information available, *n* = 3 453

**Regular iron supplementation

DTP, Diphteria, Tetanus and Pertussis vaccination ≥ 3 times; Polio vaccination ≥ 2 time

Adjusted for mother’s smoking and use of antibiotics during pregnancy

## Discussion

In this prospective study of newborns from 1966 in the two northernmost provinces and their mothers in Finland, we found that IBD is more common than previously reported in any population [24] and that maternal smoking was associated with the later development of CD but not UC in the offspring. On the other hand, DTP, and polio vaccinations during the first year of life did not increase the risk for CD or UC. Regular iron supplementation during the first year of life was negatively associated with later development of CD.

It has been shown that a first-degree relative affected with IBD has an impact on the future risk of developing IBD, as was the case in this study concerning fathers with UC [6]. Smoking has long been associated with IBD, especially CD [25]. Current smokers have an increased risk of being diagnosed with CD and tend to have a more complicated course of the disease [25]. According to our multivariate analysis, smoking during pregnancy was associated with a high risk of CD in the offspring if mothers continued smoking more than 10 cigarettes/day during pregnancy. It has been reported that maternal smoking may reduce the risk of IBD, both CD and UC [26], but several other studies have reported an increased risk [8, 27–29]. However, the mechanism through which smoking during pregnancy may influence the risk of contracting IBD remains unclear. One explanation might be that smoking is known to alter the intestinal microbiome and reduce its diversity [30]. Thus, if the mother’s intestinal bacterial flora has experienced unfavorable changes because of smoking, this may also reflect the newborn’s early development of the intestinal microbiota [31]. There is also evidence that maternal smoking changes offspring DNA methylation, which may lead to an increased risk of inflammatory bowel disease [32].

Antibiotic treatment during pregnancy has been reported to increase the risk for IBD overall but not according to disease subtype [33]. Similar results were also found in a recent study reporting that antibiotics during pregnancy were associated with an increased risk of IBD and CD but not antibiotics during infancy [34]. In this study, we found that antibiotics during pregnancy were associated with CD in the unadjusted analysis but we could not confirm this in the adjusted analysis possibly due to power issues. Other studies have reported an association between IBD and early childhood antibiotic consumption but not antibiotic use during pregnancy [14, 35].

The results from breastfeeding in this study further support previous findings that there is an inverse association between breastfeeding and developing IBD later in life [33]. A recent meta-analysis revealed this protective effect in both UC and CD patients [16], but in our univariate analysis, it was significant in CD patients. The possible underlying mechanisms behind this protective effect are most likely multifactorial. Breastfeeding likely modifies the intestinal microbiome in a beneficial way during infancy and has a vital impact on the development of innate mucosal immunity [36].

Interestingly, iron supplementation during the first year of life seems to protect against CD. In 1966–1967 neither iron nor vitamin D was added in foodstuff, and it was customary to recommend supplementation during child health center visits. The Majority of the present study subjects received supplementation of both iron (52%) and vitamin D (79%, respectively). This finding has not been reported in earlier studies. Oral administration of iron reportedly affects bacterial communities in the intestine and the metabolomic landscape in patients with IBD, but these effects could not be examined in the present study [37, 38]. The protective effect of iron was surprising because several previous studies in older children suggested that changes in the intestinal microbiome induced by oral iron medication may not necessarily be beneficial. For example, in a study involving African children aged 6 to 14 years and living in a rural area of the Ivory Coast, those children who received iron-fortified biscuits for 6 months had an unfavorable ratio of fecal enterobacteria to bifidobacteria and lactobacilli and elevated fecal calprotectin compared to those in a control group [38]. Similarly, a study of 6-month-old Kenyan infants who received iron-fortified maize porridge for 4 months reported a greater prevalence of pathogenic intestinal bacteria, including Salmonella, Clostridium spp. and *Escherichia coli* spp., and elevated fecal calprotectin levels than did infants in the control group [39]. However, no association with IBD was found. In a study of adolescents and young adults who had IBD, oral iron therapy was well tolerated and did not increase disease activity [40]. Although oral iron has been associated with negative effects on the gut microbiome, we found that oral iron supplementation during the first year of life was associated with a lower risk of CD, and this needs to be confirmed.

It was reassuring that DTP and polio vaccinations are not harmful and may even be beneficial in terms of the risk for IBD. Immunization against mumps was previously observed to lower the risk of UC [13]. One study showed an increased risk for IBD related to Bacillus Calmette–Guérin (BCG) vaccination [12]. This was not possible to investigate in our study because there was only one patient with IBD who did not receive BCG vaccination; however, in a Danish cohort study, BCG vaccination had a slight protective effect if given before 4 months of age [41]. Our findings further suggest that it is unlikely that vaccination increases the risk for IBD. Other data indicate that vaccinations do not increase the prevalence of other autoimmune diseases or allergies [42]. The mechanisms underlying the possible protective effect are unknown. We speculate that the developing immune system needs targets to not act inappropriately in regard to contact with microbial species later in life, i.e., the hygiene hypothesis [43].

The urban environment has been investigated in many studies, and a meta-analysis concluded that there is a positive association between living in an urban area and both CD and UC [18]. In our study, however, this difference was not associated with the development of CD or UC. On the other hand, we only evaluated this risk factor at the time of birth i.e., we had no data where these patients with IBD lived later when their disease was diagnosed. Similarly, animal contact did not alter IBD risk, as was reported in a cohort study from Slovakia [44] and in a recent meta-analysis [45]. These findings may be related to the fact that even the largest city or town of the NFBC area (Oulu) had approximately 78 000 inhabitants in 1966, and it was not rare to see farm animals; for instance, livestock inside city limits only a few kilometers from town centers. However, in recent decades, there has been a rapid urbanization in Finland, as has a change to a higher intake of ultra-processed food, which has been associated with an increased risk of IBD [46, 47]. High social class has been linked to increased IBD risk [13, 48], but in our study, we did not observe any effect of social class. This may have been because social class in our cohort was defined as parents’ occupation rather than true household income.

The main strength of the study is the study population, NFBC1966, and its systematic follow-up of more than approximately 50 years. The data used in this study were prospectively collected during pregnancy, at labor and at the first-year visit to the child health center, which diminishes any recall bias. The diagnosis of IBD is reliable; most of the diagnosed cases were confirmed in the SII drug reimbursement database, with only 2 cases with no ongoing reimbursement in 2016, and these diagnosed cases are very accurate because they are based on chronic and/or relapsing courses of disease found by endoscopy and histology. One study analyzed the diagnostic accuracy of pediatric IBD in Finland, and among 50 randomly selected patients, there was only one suspicious case [49]. Furthermore, most patients in this study were diagnosed in adulthood after 20 to 30 years of age with repeated control visits according to hospital records, and it is likely that the diagnosis of IBD was more accurate since endoscopic procedures became more widely used at that age.

This study has several limitations. First, although there was a high prevalence of IBD, the number of patients (154) was relatively low, and data from all the measured variables were not available for all the individuals. However, also in a recent study from Finland, increasing incidence and prevalence of IBD were found. Incidence increased from 28 to 48 and prevalence from 376 to 972 per 100,000, respectively between 2000 and 2020 [50]. They also observed that the incidence rate ratio was highest in the northern Oulu University District (1.27 for CD and 1.32 for UC) where our study was initiated. Subjects in the present study were all born in 1966 and are now in the middle age. In the Finnish study named above, the increase in the incidence was most notable in the elderly [50]. Second, although most individuals available at the time of the 46-year questionnaires (67.5%) provided informed consent for the analysis of the data, we were not able to obtain information from the whole original cohort (12 058 children). Additionally, the incidence of IBD in patients who provided informed consent was slightly greater than that in patients who did not, which may result in selection bias.

In conclusion, IBD was common in this population from Northern Finland. Smoking during pregnancy was associated with a high increased risk for CD in the offspring. Breastfeeding and oral iron supplementation during the first year of life were associated with decreased risk for CD later in life. We also conclude that the routine vaccination program in Finland was not associated with increased risk for IBD.

## Data Availability

The data underlying this article were provided by NFBC1966 with permission. The data will be shared upon request to the corresponding author with the permission of NFBC1966.
